# A Silver Fluoride Intervention to Improve Oral Health Trajectories of Young Indigenous Australians: Protocol for a Cluster Randomized Controlled Trial

**DOI:** 10.2196/48558

**Published:** 2023-11-02

**Authors:** Joanne Hedges, Gustavo Hermes Soares, Yvonne Cadet-James, Zell Dodd, Sinon Cooney, James Newman, Murthy Mittinty, Sanjeewa Kularatna, Priscilla Larkins, Roman Zwolak, Rachel Roberts, Lisa Jamieson

**Affiliations:** 1 Australian Research Centre for Population Oral Health Adelaide Dental School The University of Adelaide Adelaide Australia

**Keywords:** clinical trial, community, dental caries, Indigenous Australian, intervention, silver fluoride intervention

## Abstract

**Background:**

Indigenous Australian children and adolescents experience profound levels of preventable dental disease. The application of silver fluoride (AgF) to active dental caries is a noninvasive alternative to traditional dental treatment approaches. There is particular utility among Indigenous children and young people with dental fear, who may not have access to timely or culturally safe dental service provisions.

**Objective:**

The aims of this study are to: (1) assess levels of active dental caries among Indigenous children and young people in 6 Australian states and territories; (2) determine if an AgF intervention reduces levels of active disease over 12-24 months; (3) measure the impact of improved oral health on social and emotional well-being (SEWB) and oral health-related quality of life; and (4) calculate the cost-effectiveness of implementing such an initiative.

**Methods:**

The study will use a 2-arm, parallel cluster randomized controlled trial design. Approximately 1140 Indigenous children and youth aged between 2 and 18 years will be recruited. Each state or territory will have 2 clusters. The intervention group will receive the AgF intervention at the start of the study, with the delayed intervention group receiving the AgF intervention 12 months after study commencement. The primary outcome will be the arrest of active carious lesions, with arrested caries defined as nonpenetration by a dental probe. Secondary outcomes will include SEWB, oral health-related quality of life, and dental anxiety, with covariates including dental behaviors (brushing and dental visits). Effectiveness measures for the economic evaluation will include the number of children and young people managed in primary oral health care without the need for specialist referral, changes in SEWB, the numbers and types of treatments provided, and caries increments.

**Results:**

Participant recruitment will commence in May 2023. The first results are expected to be submitted for publication 1 year after a 24-month follow-up.

**Conclusions:**

Our findings have the potential to change the way in which active dental disease among Indigenous children and young people can be managed through the inclusion of specifically tailored AgF applications to improve dental health and SEWB delivered by Indigenous health care workers. Desired impacts include cost savings on expensive dental treatments; improved SEWB, nutrition, social, and learning outcomes; and improved quality of life for both children and young people and their caregivers and the broader Indigenous community. The AgF application could be easily implemented into the training program of Indigenous health workers and yield critical information in the management armamentarium of health and well-being recommendations for Australia’s First Peoples.

**International Registered Report Identifier (IRRID):**

PRR1-10.2196/48558

## Introduction

### Young Indigenous Australian Oral Health

Indigenous Australian children and adolescents experience profound levels of preventable dental disease [1]. These have an impact on Indigenous children’s and young people’s ability to eat, speak, socialize, and learn. It is a leading cause of child malnutrition, poor quality of life, and nonoptimal social and emotional well-being (SEWB) [2]. Trajectories of dental disease among Indigenous children and adolescents are increasing at a rate far greater than for non-Indigenous children and adolescents, with severe cases requiring care under a hospital-based general anesthetic [3]. Poor oral health in childhood is the leading cause of poor oral health in adulthood [4] and has associations with other systemic conditions, including diabetes, cardiovascular disease, and chronic kidney disease [5].

### Dental Treatment Under General Anesthesia

Provision of dental care to young children can pose many challenges because of their emotional and physical development and lack of cooperation in the dental chair. The issues are multiplied for Indigenous children in rural and remote locations, where access to dental care is severely limited. Hospital-based treatment under general anesthesia is becoming an increasingly used mode of dental care for Indigenous children, with Australian Institute of Health and Welfare statistics showing that the rate of Indigenous Australian child admissions for dental treatment is twice that of non-Indigenous children [6]. However, dental treatment under general anesthesia does not prevent the occurrence of new dental decay, with children frequently readmitted for hospital-based dental general anesthesia after initial treatment [7]. Recent reports also suggest that oral rehabilitation under general anesthesia does little to alleviate dental fear or change noncooperative behavior and may, in fact, heighten these characteristics [8,9]. Moreover, comprehensive dental care under general anesthesia is not without risk, including the potential for long-term adverse neurodevelopmental effects [10-12]. Dental general anesthetics are extremely expensive for the taxpayer, the Australian health care system, and the community more broadly, and require considerable time and financial investments from carers (to transport the child to the hospital, stay overnight, ensure fasting before the operation, etc). The estimated mean cost of dental general anesthetics for Indigenous children is substantially higher than the cost of care for non-Indigenous children [10]. Treatment and preventive approaches that can be undertaken in Indigenous primary care settings to reduce the number of Indigenous children undergoing dental general anesthetics are thus urgently required.

### Interventions to Improve Indigenous Children’s Oral Health

In the past decade, there have been 4 published interventions aimed at reducing the burden of dental caries among Indigenous Australian children. Slade and colleagues [13] conducted a cluster randomized controlled trial of a dental health program that included the application of fluoride varnish to the teeth of Indigenous children aged between 18 and 47 months and dental education in 30 remote communities in the Northern territory. Although the study team reported reduced experience of dental disease among the intervention group compared with the control group, overall levels of dental disease at 2-year follow-up were extremely high, with 94% of children having experienced caries. Jamieson et al [14] conducted an early childhood caries intervention among Indigenous children and their families in South Australia, commencing during pregnancy and when children were aged 6, 12, and 18 months old. The intervention comprised dental care during pregnancy, fluoride varnish application to the teeth of children, anticipatory guidance, and motivational interviewing. At 5-year follow-up, children in the immediate intervention group had significantly less decay than those in the delayed intervention group (who received intervention at ages 2 to 3 years), but 47% of children still had experience of dental disease (Jamieson et al [14]). Smith et al [15] tested the effectiveness of a dental education program among young Indigenous children and their families from 8 Aboriginal Community Controlled Health Organizations (ACCHOs) in New South Wales. They reported that children in the education program had fewer dental caries at age 30 months than a comparator group drawn from the community, but the study did not follow standard randomization processes, meaning the conclusions are weakened (Smith et al [15]). Roberts-Thomson et al [16] used a silver fluoride (AgF) intervention among remote-dwelling Indigenous children and reported that children exposed to the intervention had less dental pain, dental extractions, use of antibiotics for dental infections, and fillings than children not exposed to the intervention. AgF was reportedly well accepted, easy to use, and at least as effective as conventional dental care (fillings and extractions). There were limitations, however, including a lack of robust randomization, only 2 trial sites included, only primary molar teeth included, a large loss to follow-up, and outcome measures not including specific assessments of dental caries or any patient-reported outcome measures such as oral health-related quality of life (OHRQoL), SEWB, and dental anxiety. As such, it has not been able to influence policy. A study that has more rigor (ie, is properly randomized), has a larger number of study sites (ie, more power in a statistical sense), uses explicit measures of dental caries (such as tooth surfaces with active dental decay), includes all present teeth, and comprehensively measures changes to SEWB, quality of life, and anxiety is urgently needed. Only then can we realistically expect policy changes, with the ultimate goal being to introduce the treatment for adults as well.

### What is Silver Fluoride and How Does it Prevent Dental Caries?

AgF is a cariostatic agent clinically applied to manage active dental caries to prevent further progression of disease. The fluid comprises 2 key therapeutic ingredients: silver and fluoride. The fluoride helps replenish the fluoride reservoir in the enamel (the outermost surface of the tooth) to ensure a robust crystalline structure and to promote the remineralization of carious lesions. Formulations of AgF at 38% present the highest concentration of fluoride (44,800 ppm F) among the available remineralizing agents—nearly twice the concentration of professionally applied fluoride varnish (22,600 ppm F) [17]. The silver acts as an antimicrobial agent, altering the composition of cariogenic biofilm within the dentine tubules of treated lesions (Sulyanto et al [18]). In contact with decalcified dentine, AgF forms insoluble silver microwires that block dentine tubules and contribute to the increased hardness of tissues [19]. AgF also has an inhibitory effect on matrix metalloproteinases involved in the degradation of collagen [20]. Unlike other modes of treatment for active dental caries, AgF may be applied by nondentally qualified staff, meaning the cost-benefit ratio is substantial [21]. Although it has been around for at least fifty years, the uptake of AgF has been limited because, under the old formulation, the active ingredient (silver) stained decalcified soft dentine black. However, the application of a potassium iodide solution immediately after the placement of AgF has been reported to reduce staining and improve aesthetic outcomes [22,23]. The World Health Organization has recently included AgF on the list of essential medicines for children [24].

### Why is Economic Evaluation Important?

In Australia, the Pharmaceutical Benefit Advisory Committee and Medical Services Advisory Committee require evidence of cost effectiveness for their reimbursement decisions. As such, it is important to conduct an economic evaluation for any health intervention to ensure financial sustainability in addition to clinical effectiveness. Moreover, to convince state and federal health services to sustain the funding of a new health intervention, it is imperative to prove the cost-effectiveness of the program. In the broader scientific community, there is now an expectation that clinical trials evaluating medicines and procedures should additionally assess the economic value of such interventions [25]. This expectation reflects both interest in economic information for new technologies and the regulatory and reimbursement regimes that require evidence of economic value along with clinical efficacy before implementing policy change. Nowhere is this more critical than in the field of young Indigenous Australian dental care. Indigenous Australians experience a disproportionate burden of disease, which translates into a 30% higher cost of health care delivery per capita for Indigenous populations compared to the general population [26]. Any demonstration of the cost-effectiveness of a dental care intervention over standard care is going to be of benefit to not only Indigenous children receiving the intervention but also to taxpayers contributing to the health care budget. To the best of our knowledge, there have been few evaluations of the cost-effectiveness of child dental caries interventions. The only one within the Indigenous child dental space was evaluated by Kularatna et al [27], with economic evaluations of dental health interventions per se being scarce.

Most ACCHOs in regional and remote locations in Australia are unable to provide dental care, yet they recognize that these services are critically important [28]. AgF can be applied by nondental personnel in home environments with minimal cost and equipment. This means AgF implementation, uptake, and cultural acceptability are superior to many other expensive health service models delivered by non-Indigenous staff.

The aims of this study are to (1) assess levels of active dental caries among Indigenous children and young people in 6 Australian states and territories; (2) determine if an AgF intervention reduces levels of active disease over 12-24 months; (3) measure the impact of improved oral health on SEWB and OHRQoL; and (4) calculate the cost-effectiveness of implementing such an initiative.

## Methods

### Overview

This is an Indigenous-led project conducted in partnership with ACCHOs across 6 states or territories in Australia. Meaningful and respectful engagement with ACCHOs ensures Indigenous self-determination and culturally appropriate research practices for the context of each Indigenous community throughout all stages of the study.

### Study Design

**Figure 1:**
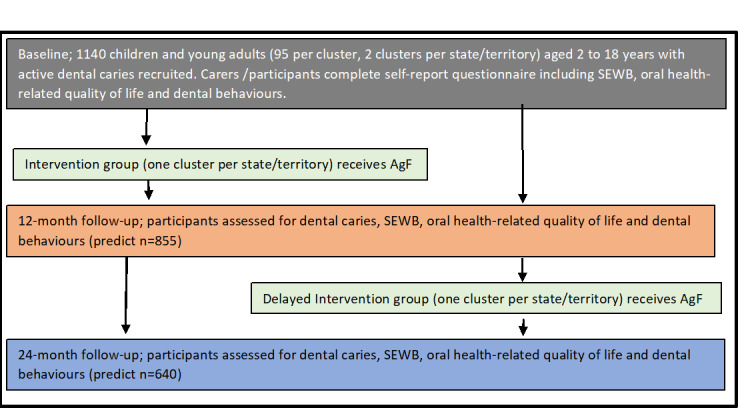
Study plan schema. AgF: silver fluoride; SEWB: social and emotional well-being.

The study will use a 2-arm, parallel, matched-paired cluster randomized controlled trial design with 2 study sites (clusters) in each partnering state and territory (Figure 1). The intervention group will receive the AgF intervention at the start of the study, with the delayed intervention group receiving the AgF intervention 12 months after study commencement.

### Inclusion Criteria

Indigenous children and young adults aged between 2 and 18 years with active carious lesions (nonpulpally involved) in the primary or permanent dentition residing in selected study clusters will be eligible. Caregivers or participants who are unable to provide written informed consent, participants with a history of allergy to silver, or those presenting oral ulceration will be excluded. At the tooth level, symptomatic or pulpally involved teeth (pulp exposure, presence of an abscess or fistula, obvious discoloration indicative of pulp necrosis, or premature hypermobility) will be excluded from receiving the intervention.

### Randomization

The randomization schedule will be developed by biostatisticians at the Australian Research Centre for Population Oral Health, who will not be involved in the trial, using a computer-generated block-randomized algorithm. The random allocation will be 1:1 per state and territory; that is, 1 cluster per state or territory will be randomly allocated as the intervention site, leaving the other cluster to be the delayed intervention site. Randomization will be done before baseline fieldwork. Field staff will have no access to the randomization algorithm, thus allowing the randomization process to be audited.

### Participant Recruitment

We will adopt recruitment strategies that have been successfully applied in our wide body of research with Indigenous communities across Australia and that build upon the extensive community consultation processes that have already been undertaken for the proposed study. We will employ local Indigenous people as research assistants to continue to facilitate community engagement and participant recruitment. These research assistants will, in turn, be guided by senior Indigenous project officers. All participants at baseline will receive a clinical dental examination by a calibrated dental examiner, with participants aged 15 years or older or caregivers asked to complete a baseline questionnaire containing items on SEWB, oral health-related quality of life, dental behaviors, and sociodemographic characteristics. Participants in the delayed intervention cluster will be able to access standard dental care during the first 12 months of the study at their own volition.

### Reimbursement for Time

As per our standard procedures for participants involved in health research projects, all participants will receive an Aus $20 (US $15) gift voucher as a reimbursement for their time at both the initial recruitment and 12- and 24-month follow-ups. Each participant will additionally receive a sample bag of oral and general health-related items at baseline, including fluoridated toothpaste and toothbrushes.

### Intervention

After cleaning teeth with dry gauze and drying the cavities with cotton pellets, dentally qualified research officers will apply 38% AgF (Riva Star Aqua Step 1, Southern Dental Industries Ltd) to sites of active caries in the primary and permanent dentition using a microbrush applicator and keeping the solution in contact with the lesion for 60 seconds. No attempts will be made to remove soft dentine. Potassium iodide (Riva Star Aqua Step 2, Sothern Dental Industries Ltd) will be applied immediately after Riva Star Aqua Step 1 until the white precipitate is clear, signaling that all free silver ions have reacted with the potassium iodide. Petroleum jelly will be applied to the lips and gingival mucosa to avoid direct contact of AgF with soft tissues. Participants will be instructed to refrain from drinking, eating, or rinsing their mouths for at least thirty minutes. A single AgF application will occur at the baseline visit for the immediate intervention clusters and at the 12-month follow-up for the delayed intervention clusters. Any participants requiring additional dental treatment at the baseline, 12-month, or 24-month follow-ups will be referred for standard dental care to the local dental service provider.

### Outcomes

The primary outcome will be the arrest of carious lesions in the dentition, with arrested caries defined as nonpenetration by a dental probe. Secondary outcomes will include SEWB, OHRQoL, dental anxiety, and dental behaviors. A SEWB measure has been developed by our team based on community consultation, item polling, and a theoretical framework that identified six core SEWB domains for Indigenous Australian youths: (1) feeling empowered; (2) feeling strong in my body; (3) feeling loved and safe; (4) feeling resilient; (5) feeling strong in my mind; and (6) feeling strong in my identity. OHRQoL will be assessed using the Caries Impacts and Experiences Questionnaire [29], which we have validated and successfully implemented in other studies involving Indigenous Australian children [30]. Dental anxiety will be assessed using the Child Dental Anxiety and Scale [31], modified to capture anxiety from adolescent participants, which we have also validated and implemented in studies involving Indigenous children [30]. Behaviors will be assessed using items from the 2012-2014 National Child Oral Health Survey [1]. These will include participant age when commenced tooth brushing, tooth brushing frequency, use of fluoride toothpaste, last time participant saw an oral health care provider, usual reason for participant to visit an oral health care provider, and consumption of sugar-sweetened beverages and foods.

Effectiveness measures for the economic evaluation will include the number of children and young people managed in primary oral health care without the need for specialist referral, changes in SEWB, the numbers and types of treatments provided, and caries increments. A cost-utility analysis will be undertaken using the recently developed dental caries utility index for adolescents [32] and ECOHIS-4D to measure the utility values of the health states experienced by children and young people [33].

### Oral Epidemiological Examinations

Caries experience will be collected through standardized oral epidemiological examinations by calibrated dental examiners. Didactic and clinical training for the examination teams will be conducted before baseline data collection, with refresher sessions provided during the fieldwork phase. Examinations will be in participants’ homes, ACCHOs, schools, or wherever the caregiver, child, or young person feels most comfortable. All examiners will be tested in the field against the senior trainer to estimate interexaminer reliability. Intraclass correlation coefficients for caries assessment scores will be used to assess reliability.

### Sample Size Calculation

Based on the literature, in which approximately 81% of carious lesions were estimated to have been arrested with AgF [34], the community intracluster correlation was calculated as 0.09. Using these parameters at 80% power, α=.05, and 12 clusters available (6 clusters per treatment arm), the estimated sample size required is 320 in each arm. Allowing for a 25% loss to follow-up each year, the estimated sample size is 570 per arm, or 95 children and adolescents per cluster (rounded up). Sample size estimations were undertaken on STATA 15 (StataCorp) using the commands for cluster randomized trials for proportions of caries arrest.

### Data Analysis

The trial has been registered in the Australian and New Zealand Clinical Trials Registry (ACTRN12622001066774p). All analysis will be reported according to CONSORT (Consolidated Standards of Reporting Trials) guidelines. Data analysis will use intention-to-treat approaches, with standard imputation measures used to manage missing data. The analysis plan for each aim is as follows.

Aim 1 (severity of dental disease in young Indigenous Australians): the severity of dental disease will be calculated using the decayed, missing, and filled tooth surface index (dmfs/DMFS) for the primary and permanent dentition. The 2014-2016 National Child Oral Health Survey will enable comparison with both general and Indigenous population-level estimates [1]. General analysis will comprise the chi-square and 1-tailed Student *t* tests within the study sample and a nonoverlapping 95% CI when comparing with population estimates.

Aim 2 (changes in dental disease following AgF intervention): the trial will be analyzed on an intention-to-treat basis according to group allocation. Generalized linear mixed models will be performed to determine differences in the primary outcome (arrest in dental caries) between the 2 groups and over time when the outcome is continuous. For binary outcomes, general estimating equations with the log binomial family will be used [35].

Aim 3 (economic evaluation of the intervention): the cost-effectiveness of the intervention will be estimated using standard approaches from a health provider perspective. Effectiveness measures will include quality of life and utility indicators. A literature review will inform long-term cost savings to the health system by improving Indigenous children’s and young people’s experiences of dental disease. The usual method of determining the cost effectiveness of a health intervention is by estimating the incremental cost effectiveness ratio, the ratio between the incremental cost and the incremental outcome. Incremental cost is the cost difference between the intervention and usual care, while incremental outcome is the outcome difference between the intervention and usual care. The economic evaluation will compare any incremental costs of the intervention (costs accrued in the intervention arm compared to those in the delayed intervention arm) to the full list of incremental primary and secondary outcome endpoints, all expressed in their natural units of measurement. Costs will be measured from activity data with pathway analysis to fully specify all activities in both the immediate and delayed intervention arms. Resource use will be obtained from research and provider records. Measured resource use will be valued using both existing estimates of the costs of each unit of resource use from market prices and the Medicare fee rates. Intervention costs will exclude research project costs. Standard discounting will be applied to both costs and outcomes, with uncertainty in these data subjected to sensitivity analyses.

### Ethical Considerations

Ethics approval (number 04-22-1013) has been obtained from the Aboriginal Health Council of South Australia Human Research Ethics Committee, the University of Adelaide Human Research Ethics Committee, the Human Research Ethics Committee of the Northern Territory Department of Health and Menzies School of Health Research, the Far North Queensland Human Research Ethics Committee, the Aboriginal Health and Medical Research Council of New South Wales Human Research Ethics Committee, the Australian Institute of Aboriginal and Torres Strait Islander Studies Human Research Ethics Committee, and the Western Australian Aboriginal Health Ethics Committee. Before being recruited, all participants will be required to sign an informed consent form, which includes consent for the authors to publish the findings in the peer-reviewed scientific literature. All authors are named investigators on the project; they all contributed to the intellectual input of the study design and in writing this protocol.

## Results

Participant recruitment for this study commenced in May 2023, and up until September 2023, around 540 children and young adults have been recruited. We anticipate the first results to be submitted for publication 2 years following initial recruitment.

## Discussion

### What are the Views of the Indigenous Community About the Study?

Feedback from multiple Indigenous participants in our current studies reveals that poor access to dental services is a key barrier to achieving optimal health. Many participants should be in the prime of their lives, yet ongoing concerns about their oral health are impacting important life decisions (eg, shame about poor oral health prevented one from singing in a television documentary, while embarrassment about bad breath prevented another from attending a job interview). These inequities begin in early childhood, with Indigenous children as young as 2 years old in some communities requiring all teeth to be removed under a hospital-based general anesthetic because of advanced dental decay.

### Why is This Study Important?

Despite the unacceptable poor oral health experienced by many Indigenous children and young people in regional and remote locations, there have been few interventions developed using culturally safe methodologies for improving oral health using low-cost approaches that are non–resource-intensive. Consequently, the management arsenal available to reduce the incidence of active dental decay among Indigenous children and young people that does not involve extensive restorative care (needles, drilling, and filling) is limited. The proposed study will investigate a novel use (AgF) for a potentially widely available public health service with which every health professional can become familiar. Currently, AgF is the only nonsurgical therapeutic option for the management of active dental caries. Our results may provide evidence for decision makers to make informed resource allocations in Indigenous oral health, which impacts both SEWB and health-related quality of life. This is especially necessary in the ACCHO sector.

### Strengths and Limitations

This study will test the hypothesis that Indigenous children and young people in 6 Australian states and territories can be provided with appropriate dental care in local Indigenous settings using the AgF approach without specialist intervention and avoiding the need for treatment under hospital-based general anesthesia. This study has six major strengths: (1) it will evaluate the and severity of dental disease in Indigenous children and young people in 6 states and territories; (2) it will test the efficacy of a simple and culturally safe AgF initiative, which could be easily implemented into the training program of Indigenous Health Workers to be included in their remit of care; (3) we will have capacity to comprehensively assess the cost-effectiveness of the intervention, meaning uptake into clinical guidelines will be much swifter than if no cost-effective analysis was undertaken; (4) the intervention will likely lead to SEWB improvements in Indigenous children and young people; an important strategic priority given current commitments to improving child health and well-being, with the Australian Government launching, in October 2021, the world’s first National Children's Mental Health and Well-Being Strategy [36]; (5) we have engaged and consulted considerably with participating ACCHOs, using their feedback to shape the research questions and; (6) our researchers are world-class experts in their respective specialties and have experience working with Indigenous child and adolescent populations. Limitations include operationalizing a national-level trial while COVID-19 is still present and assessing active dental caries by clinical examination only (no use of radiographs). Conducting a clinical trial in challenging contexts and following the cultural protocols of local Indigenous communities prevents us from blinding study participants.

In conclusion, our findings have the potential to change the way in which the oral health of Indigenous children and young people is managed, with desired impacts including cost savings on expensive dental treatments, improved SEWB, nutrition, social, and learning outcomes, and improved quality of life for both children and young people and their caregivers and the broader Indigenous community. This study addresses priorities raised in the National Aboriginal and Torres Strait Islander Health Plan 2013-2023 [37], particularly in regard to increasing understanding of Indigenous health issues at a community level, improving public health measures that address Indigenous health, and facilitating the collection of Indigenous health data.

## Abbreviations

ACCHO: Aboriginal Community Controlled Health Organization
AgF: silver fluoride
CONSORT: Consolidated Standards of Reporting Trials
dmfs/DMFS: decayed, missing, and filled tooth surface index
OHRQoL: oral health-related quality of life
SEWB: social and emotional well-being

## References

[1] Do LG, Spencer AJ (2016). Oral Health of Australian Children: The National Child Oral Health Study 2012-14.

[2] Jamieson LM, Paradies YC, Gunthorpe W, Cairney SJ, Sayers SM (2011). Oral health and social and emotional well-being in a birth cohort of Aboriginal Australian young adults. BMC Public Health.

[3] Jamieson LM, Armfield JM, Roberts-Thomson KF (2006). Oral health inequalities among Indigenous and nonindigenous children in the Northern Territory of Australia. Community Dent Oral Epidemiol.

[4] Isaksson H, Alm A, Koch G, Birkhed D, Wendt LK (2013). Caries prevalence in Swedish 20-year-olds in relation to their previous caries experience. Caries Res.

[5] Handsley-Davis M, Jamieson L, Kapellas K, Hedges J, Weyrich LS (2020). The role of the oral microbiota in chronic non-communicable disease and its relevance to the Indigenous health gap in Australia. BMC Oral Health.

[6] (2014). Oral Health and Dental Care in Australia: Key Facts and Figures Trends 2014.

[7] Tahmassebi JF, Achol LT, Fayle SA (2014). Analysis of dental care of children receiving comprehensive care under general anaesthesia at a teaching hospital in England. Eur Arch Paediatr Dent.

[8] Zhou F, Zhang S, Ma W, Xiao Y, Wang D, Zeng S, Xia B (2022). The long-term effect of dental treatment under general anaesthesia or physical restraints on children's dental anxiety and behaviour. Eur J Paediatr Dent.

[9] Cantekin K, Yildirim MD, Cantekin I (2014). Assessing change in quality of life and dental anxiety in young children following dental rehabilitation under general anesthesia. Pediatr Dent.

[10] Alsharif AT, Kruger E, Tennant M (2015). A population-based cost description study of oral treatment of hospitalized Western Australian children aged younger than 15 years. J Public Health Dent.

[11] Lee HH, Milgrom P, Starks H, Burke W (2013). Trends in death associated with pediatric dental sedation and general anesthesia. Paediatr Anaesth.

[12] Ing C, DiMaggio C, Whitehouse A, Hegarty MK, Brady J, von Ungern-Sternberg BS, Davidson A, Wood AJJ, Li G, Sun LS (2012). Long-term differences in language and cognitive function after childhood exposure to anesthesia. Pediatrics.

[13] Slade GD, Bailie RS, Roberts-Thomson K, Leach AJ, Raye I, Endean C, Simmons B, Morris P (2011). Effect of health promotion and fluoride varnish on dental caries among Australian Aboriginal children: results from a community-randomized controlled trial. Community Dent Oral Epidemiol.

[14] Jamieson LM, Smithers LG, Hedges J, Aldis J, Mills H, Kapellas K, Lawrence HP, Broughton JR, Ju X (2019). Follow-up of an intervention to reduce dental caries in indigenous Australian children: a secondary analysis of a randomized clinical trial. JAMA Netw Open.

[15] Smith L, Blinkhorn F, Moir R, Blinkhorn A (2018). Results of a two year dental health education program to reduce dental caries in young Aboriginal children in New South Wales, Australia. Community Dent Health.

[16] Roberts-Thomson KF, Ha DH, Wooley S, Meihubers S, Do LG (2019). Community trial of silver fluoride treatment for deciduous dentition caries in remote Indigenous communities. Aust Dent J.

[17] Gao SS, Zhang S, Mei ML, Lo ECM, Chu CH (2016). Caries remineralisation and arresting effect in children by professionally applied fluoride treatment - a systematic review. BMC Oral Health.

[18] Sulyanto RM, Kang M, Srirangapatanam S, Berger M, Candamo F, Wang Y, Dickson JR, Ng MW, Ho SP (2021). Biomineralization of dental tissues treated with silver diamine fluoride. J Dent Res.

[19] Seto J, Horst JA, Parkinson DY, Frachella JC, DeRisi JL (2020). Enhanced tooth structure via silver microwires following treatment with 38 percent silver diamine fluoride. Pediatr Dent.

[20] Mei ML, Ito L, Cao Y, Li QL, Lo ECM, Chu CH (2013). Inhibitory effect of silver diamine fluoride on dentine demineralisation and collagen degradation. J Dent.

[21] (2022). Policy on the use of silver diamine fluoride for pediatric dental patients. The Reference Manual of Pediatric Dentistry.

[22] Garg S, Sadr A, Chan D (2019). Potassium iodide reversal of silver diamine fluoride staining: a case report. Oper Dent.

[23] Turton B, Horn R, Durward C (2021). Caries arrest and lesion appearance using two different silver fluoride therapies on primary teeth with and without potassium iodide: 12-month results. Clin Exp Dent Res.

[24] (2022). WHO model list of essential medicines for children - 8th list, 2021. World Health Organization.

[25] Ramsey SD, Willke RJ, Glick H, Reed SD, Augustovski F, Jonsson B, Briggs A, Sullivan SD (2015). Cost-effectiveness analysis alongside clinical trials II-An ISPOR Good Research Practices Task Force report. Value Health.

[26] (2023). Aboriginal and Torres Strait Islander health performance framework - summary report. Australian Institute of Health and Welfare.

[27] Kularatna S, Lalloo R, Kroon J, Tadakamadla SKK, Scuffham PA, Johnson NW (2020). Demonstration of high value care to improve oral health of a remote Indigenous community in Australia. Health Qual Life Outcomes.

[28] Krichauff S, Hedges J, Jamieson L (2020). 'There's a wall there-and that wall is higher from our side': drawing on qualitative interviews to improve Indigenous Australians' experiences of dental health services. Int J Environ Res Public Health.

[29] Gilchrist F, Rodd HD, Deery C, Marshman Z (2018). Development and evaluation of CARIES-QC: a caries-specific measure of quality of life for children. BMC Oral Health.

[30] Arrow P, Brennan D, Mackean T, McPhee R, Kularatna S, Jamieson L (2021). Evaluation of the ECOHIS and the CARIES-QC among an Australian "Aboriginal" population. Qual Life Res.

[31] Howard KE, Freeman R (2007). Reliability and validity of a faces version of the modified child dental anxiety scale. Int J Paediatr Dent.

[32] Hettiarachchi RM, Kularatna S, Byrnes J, Chen G, Mulhern B, Scuffham PA (2022). Development of a classification (descriptive) system for a preference-based quality of life measure for dental caries (dental caries utility index) among adolescents. J Public Health Dent.

[33] Hettiarachchi RM, Arrow P, Senanayake S, Carter H, Brain D, Norman R, Tonmukayawul U, Jamieson L, Kularatna S (2022). Developing an Australian utility value set for the Early Childhood Oral Health Impact Scale-4D (ECOHIS-4D) using a discrete choice experiment. Eur J Health Econ.

[34] Gao SS, Zhao IS, Hiraishi N, Duangthip D, Mei ML, Lo ECM, Chu CH (2016). Clinical trials of silver diamine fluoride in arresting caries among children: a systematic review. JDR Clin Trans Res.

[35] Hemming K, Taljaard M, Forbes A (2017). Analysis of cluster randomised stepped wedge trials with repeated cross-sectional samples. Trials.

[36] (2021). National children’s mental health and wellbeing strategy. National Mental Health Commission, Australian Government.

[37] (2013). National Aboriginal and Torres Strait Islander health plan 2013-2023. Department of Health and Aged Care, Australian Government.

